# Spectro-electrochemistry of guaiacol oxidation: tracking intermediates in a membrane-separated cell with *in situ* attenuated total reflectance-infrared spectroscopy†

**DOI:** 10.1039/d5fd00069f

**Published:** 2025-10-27

**Authors:** Sibylle M. K. Schwartmann, Mariangela Biggiero, Sander Deelen, Bettina Baumgartner, Bert M. Weckhuysen

**Affiliations:** a Institute for Sustainable and Circular Chemistry, Utrecht University Universiteitsweg 99 3584 CG Utrecht The Netherlands b.m.weckhuysen@uu.nl; b Van’t Hoff Institute for Molecular Sciences, University of Amsterdam Science Park 904 1098 XH Amsterdam The Netherlands b.baumgartner@uva.nl; c Scientific Instrumentation, Faculty of Science, Utrecht University 3584 CS Utrecht The Netherlands

## Abstract

Lignin, a structurally complex biopolymer, represents a promising renewable feedstock for the production of platform chemicals, including functionalized aromatic molecules. However, efficient lignin valorization remains a major challenge due to its chemical stability, structural heterogeneity, and the propensity of reactive intermediates to undergo recondensation. To overcome these barriers and gain mechanistic insight into lignin oxidation pathways, we have developed a membrane-separated, two-compartment attenuated total reflectance infrared (ATR-IR) spectro-electrochemical cell for the *in situ* monitoring of the electrochemical oxidation of lignin model compounds. Using guaiacol as a representative model compound of the β-O-4 linkage monomer, we tracked real-time spectral changes during electrochemical oxidation. Characteristic vibrational signatures revealed the depletion of guaiacol and the formation of oxidized species, including quinones, catechols, and dimers and oligomers. In contrast, control experiments conducted without membrane separation exhibited additional spectral features, suggesting the occurrence of competing side reactions under conditions of unrestricted mass transport. These results highlight the importance of proper cell design for providing mechanistic insights and demonstrate the value of *in situ* ATR-IR spectroscopy in tracking the complex electrochemical transformation of lignin-derived molecules, to offer insights critical for advancing lignin valorization strategies under mild and tunable reaction conditions.

## Abstract 2

Membrane-separated ATR-IR spectro-electrochemistry enables real-time tracking of guaiacol oxidation intermediates, revealing pathways and suppressing side reactions.

## Graphical abstract

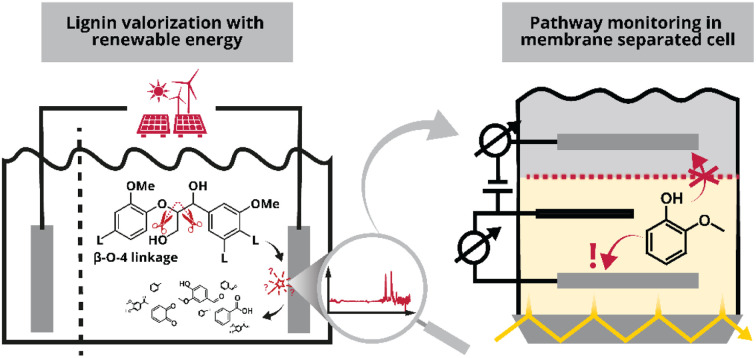

## 1. Introduction

**Fig. 1:**
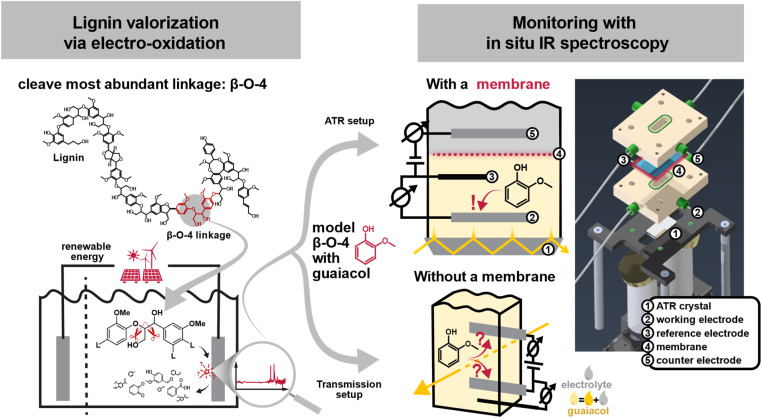
Lignin, a widely available yet underutilized complex biomolecule, has the potential to serve as a renewable feedstock for aromatic base chemicals, provided that efficient and selective depolymerization methods can be developed to yield its phenylpropanoid units. Electro-oxidative valorization offers a promising approach, enabling direct utilization of renewable energy sources, while simultaneously providing energy storage capabilities. To optimize depolymerization pathways, we designed a novel two-chamber *in situ* spectro-electrochemical cell in Attenuated Total Reflection (ATR) configuration. Unlike conventional commercial cells in transmission configuration, our design incorporates a membrane that separates the working and counter electrodes, preventing unintended reactions at the counter electrode and thus potentially misleading mechanistic insights. The figure presents a schematic diagram of the cell alongside a 3D rendering used for its construction.

The transition towards a sustainable, low-carbon economy calls for renewable alternatives to fossil-based feedstocks, such as biomass and plastic waste, particularly for the production of transportation fuels as well as key chemical building blocks, such as aromatics. Lignin, a major component of lignocellulosic biomass, is one of the most abundant candidates for a renewable resource of aromatic compounds. Despite its large potential, lignin remains mainly underutilized and often discarded as industrial waste.^1–4^ However, unlocking lignin’s value requires selective cleavage of its complex, polymeric structure into useful chemical building blocks. Lignin is an amorphous, highly heterogeneous polymer composed of phenylpropanoid units linked *via* a variety of bonds, primarily aromatic and aliphatic ethers. Among these, the β-O-4 ether linkage is the most prevalent and is often targeted in lignin depolymerization strategies (Fig. 1).^2,5,6^ Effective lignin valorisation must also balance the extent of bond cleavage (*i.e.*, depolymerization), prevent the undesired re-condensation (*i.e.*, repolymerization), and selective functionalization of reactive intermediates to yield target products or to enable further downstream processing.^4,5,7,8^ A wide variety of approaches have been explored, ranging from homo- and heterogeneous catalysis, photo- or electrocatalytic methods to thermal depolymerization techniques or enzymatic and biotechnical procedures.^5,7–10^

Among these, electrochemical valorisation is especially promising to foster the renewable energy transition. It directly integrates renewable energy and thereby offers a dual role as a green chemical production method and a form of energy storage. Furthermore, electrochemical methods typically operate under mild reaction conditions and could enable easy control over reaction selectivity and efficiency through the regulation of applied potential and current.^10,11^ However, the intrinsic complexity and stability of lignin poses significant challenges, in addition to poor product selectivity, high catalyst costs, and issues related to lignin condensation. Overcoming these hurdles requires the development of economically viable pathways for converting lignin into value-added fuels and chemicals.^1,3,6,7^ To overcome these obstacles, a fundamental understanding of the underlying reaction mechanisms is essential. Because the structural heterogeneity of native lignin makes direct mechanistic studies difficult, researchers often turn to well-defined model compounds that mimic key linkage motifs, most notably the β-O-4 ether bond with different degrees of molecular complexity. These models provide a controlled platform for systematically probing reaction conditions, intermediate formation, and product distribution.^2^

*In situ* infrared (IR) spectroscopy is one of the ideal analytical methods to study these chemical conversions, thanks to its non-destructive nature and high chemical selectivity *via* the access to the vibrational fingerprint region. Its ability to track reactions in real time makes it especially suited to monitor transient species. However, most commercial *in situ* IR electrochemical cells lack physical separation between the working and counter electrodes, introducing artefacts into the measurements from re-oxidation or re-reduction events that can obscure their mechanistic interpretation (Fig. 1). Dunwell *et al.* addressed this limitation by designing an *in situ* ATR cell optimized for surface-sensitive investigations of water adsorption on gold electrodes.^12^ Their setup is based on a modified H-cell configuration and leverages surface-enhanced infrared absorption spectroscopy (SEIRAS) for signal amplification at the gold interface. While the design is well suited for probing interfacial phenomena, its relatively large cell volume may limit the necessary sensitivity to low-concentration intermediates present in the bulk electrolyte, potentially hindering detection of species beyond the electrode surface.

In this work, we tackle these limitations by developing a dedicated *in situ* IR cell based on an Attenuated Total Reflectance (ATR) configuration. This new, straightforward design is chemically robust, and incorporates a membrane to separate electrodes, as well as the space for a commercial reference electrode. It enables both reproducible electrochemical measurements and clear spectroscopic monitoring (Fig. 1). We applied this experimental setup to study the electrochemical conversion of guaiacol, a β-O-4 model monomer. Guaiacol is inexpensive, chemically stable, and safe to handle, making it well suited for systematic ATR-IR spectroscopy studies. During electrolysis, we observed the conversion of guaiacol monitored by the decline of its characteristic vibrational bands. Concurrently, we detected new vibrational bands that correspond to intermediates, like quinones, species frequently cited in the literature as part of early-stage reaction pathways.^13–17^ A closer comparison with reference spectra of *p*-benzoquinone and catechol revealed a strong match, further supporting our spectral assignments. Importantly, experiments conducted without separating working and counter electrode yielded many of the same key spectral features. However, there were also notable differences in the ATR-IR spectra, suggesting that removing the diffusion barrier alters the reaction environment, likely by permitting secondary reactions or undesired product crossover. These findings highlight the role of membrane separation in obtaining reliably interpretable results in electrochemical lignin model studies.

## 2. Experimental

### 2.1 Chemicals and materials

Acetonitrile (Rotisolv, HPLC grade & Baker, HPLC grade), lithium perchlorate trihydrate (LiClO_4_, Thermo Scientific, 63.0%, ACS, 68.0%), guaiacol (Thermo Scientific, 99+%), *p*-benzoquinone (Thermo Scientific, 98+%), catechol (Thermo Scientific, 99%), maleic acid (Fluka Analytical, ≥99.0%), lithium hydroxide monohydrate (Sigma Aldrich, ≥99.9%, battery grade), sulfuric acid (Rotipuran, 96%), and hydrogen peroxide (Sigma Aldrich, 30% stabilized zS) were used as received. Electrolyte solutions were prepared by weight.

### 2.2 ATR-FTIR spectroscopy and electrochemical setup

The optical measurements were conducted on a Spectrum One FTIR spectrometer (PerkinElmer) equipped with a N_2_-cooled mercury cadmium telluride (MCT) detector. For each spectrum, 64 scans were averaged (double-sided, backward–forward acquisition mode, 1 s per scan) and the resolution was set to 4 cm^−1^. The spectrometer and the sample compartment containing the ATR setup was flushed with N_2_ during the experiment. The ATR crystal was cut to 10 × 20 × 0.5 mm^3^ from double site polished 〈100〉 silicon wafers (Siegert Wafer). To achieve total reflection, the two short sides of the crystal were polished to attain a 45° angle with the crystal’s top facet giving 20 active bounces with a penetration depth of (dp = 0.53 μm, de = 10.5 μm, *n*(electrolyte) = 1.33; *n*(silicon) = 3.42 at 1500 cm^−1^).^18^ Time Based Spectrum Version 10.4.0 (PerkinElmer) was used to record the measurement of the single beam spectra. The absorbance spectra were calculated from the single beam spectra as explained in section “*In situ* spectro-electrochemical protocol” with an in-house programmed Python script.

The electrochemical measurements were conducted in the custom ATR cell as well as an H-cell. For one half-cell, the volumes were 0.77 ml and 20 ml, respectively. Current and voltage were applied and recorded with an IVIUM compactstat.h10800 potentiostat. The electrolyte was 0.1 M lithium perchlorate (LiClO_4_) in acetonitrile, with 0.1 M guaiacol added to the working electrode’s half-cell. For working and counter electrode, platinum wires were used in the ATR cell and platinum meshes were used in the H-cell. The wires had a thickness of 1 mm and 5.5 mm length exposed to the electrolyte. As reference, an Ag/Ag^+^NO_3_^−^ reference electrode was used (Ossila, non-aqueous Ag/Ag^+^ reference electrode C2015A, filled with 0.01 M AgNO_3_ in ACN). The half-cells of the working and counter electrode were separated with a lithiated Nafion 117 membrane in both the ATR and the H-cell. The membranes were prepared according to a procedure reported by Burlatsky *et al.*^19^ Electrolysis was done with chronoamperometry (CA). The operation potential was set slightly below the optimal oxidation potential according to the operation protocol detailed in section “*In situ* spectro-electrochemical protocol”.

### 2.3 Cell design

The cell was designed and fabricated in-house (see Fig. 1). The ATR crystal is placed into the beam path of the spectrometer on a small PEEK table. A gold mirror directs the IR beam to hit the facet of the ATR crystal at 45°, causing internal total reflection at the top and bottom of the ATR crystal. At the other end of the crystal, the beam exits after multiple bounces and is directed to the detector with a second gold mirror.

The electrochemical cell is placed on top of the crystal and fixed to the PEEK table with four screws. It is layered like a sandwich, with the crystal at the very bottom closing off the half-cell of the working electrode followed by a membrane which separates it from the half-cell of the counter electrode above it. Finally, the counter electrode’s half-cell is closed off on the very top with a glass lid. All parts of the cell are additionally held together by another set of four screws. Leak tightness is ensured by EPDM O-rings between each layer of the cell.

The two half-cells are flat PEEK cuboids with an oval hole punched through that creates the volume which holds the electrolyte. The electrodes are screwed in from the long side of the cuboids so that they can be contacted from the outside using crocodile clamps. The entry points of the electrodes are made leak-tight with fittings and custom ferrules. In the case of the Pt wires acting as working and counter electrode, standard 1/16′′ fittings are used, while a custom fitting for the reference electrode has been made from PEEK. The electrolyte is filled into the chambers *via* PFA 1/16′′ tubes (from Merck) fixed to the short side of the cuboids.

### 2.4 *In situ* spectro-electrochemical protocol

Before performing the electrolysis, the oxidation potential of guaiacol is established. To this end, the cell is set up with a low concentration of feedstock, 1 × 10^−4^ M, in the working chamber and pure electrolyte in the counter chamber. Then a cyclic voltammogram (CV) was recorded at 30 mV s^−1^ from 0 V to 1 V or 0.5 V to 1.5 V to avoid reduction of the feedstock. The oxidation potential can be read out from the oxidation peak in the CV. This potential is used to perform the CA, while the electrolysis is monitored with IR spectroscopy. To avoid overoxidation and electrode passivation, the potential at which the electrolysis is performed is set to 0.05 V below the oxidation potential determined from the CV. Further, to ensure detectability in the IR spectra, the concentration used in the CA is increased to 0.1 M.

After the CV, the cell and electrodes are cleaned and reassembled for the CA and the setup is left under constant nitrogen flushing of the spectrometer’s sample compartment for 4 h to reach stable conditions.

The recorded ATR-IR spectra are displayed as differential spectra using the last spectrum recorded before setting the oxidation potential as background spectrum. 128 spectra were averaged and were recorded every 70 s during the experiment. Species that are consumed appear as negative bands, while species that evolve appear as positive bands. The baseline is corrected using a linear fit applied in the region between 2680 cm^−1^ and 2000 cm^−1^.

### 2.5 *Ex situ* analytical methods

The oxidation products were analysed *ex situ* with ATR-IR spectroscopy, multiple nuclear magnetic resonance (NMR) techniques (^1^H, ^13^C, COSY, HSQC, and HMBC), as well as thin layer chromatography (TLC). To this end, the electrolysis of guaiacol was conducted in an H-cell. Feedstock concentration as well as oxidation time was kept the same as in the measurements in the ATR cell. The oxidation potential was chosen the same way as with the *in situ* ATR-IR spectroscopy measurements. After the completion of the measurement, the content of the working electrode’s cell was extracted with DCM. Subsequently, the solvent was removed under vacuum. IR spectroscopy was conducted by directly depositing the product extract onto the ATR crystal, using the blank crystal as the background spectrum. The spectrum of the unreacted feedstock extract was taken in the same manner. The NMR measurements were done on a Jeol JNM-ECZL G 400 equipped with a ROYALPROBE HFX probe measured at 400 MHz. Chemical shifts were referenced to the residual solvent signal of acetonitrile-D3. TLC plates (TLC silica gel 60 F254, Merck) were developed in hexane/benzene/acetone (40 : 30 : 30, v/v/v).^16^

## 3. Results and discussion

### 3.1 Cell characterization, validation and experimental robustness

To address the issue of re-oxidation and re-reduction artifacts commonly observed in commercial *in situ* IR cells when studying electrochemical conversions, we developed a custom, two-chamber electrochemical cell separated by a lithiated Nafion 117 membrane. The design builds on a previously reported multi-bounce ATR cell setup, which we extended with a second chamber separated by a membrane and included working, counter and reference electrodes.^18^Fig. 1 shows a render of the final design. The custom-made setup was mounted into a commercial FT-IR spectrometer for *in situ* investigations. Prior to electrochemical experiments, the permeability of the Nafion membrane against guaiacol was tested using the same setup. No permeability was observed for at least 24 h after injection, validating the membrane’s effectiveness in preventing cross-contamination between the top and bottom chambers (see ESI† for further details).

**Fig. 2:**
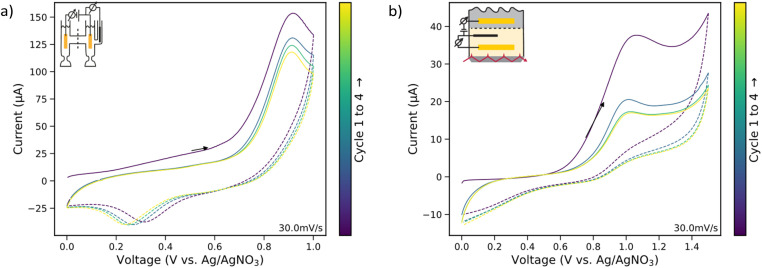
Comparison between cyclic voltammograms (CVs) obtained in (a) an H-cell and (b) in the *in situ* attenuated total reflectance (ATR) cell. Potential is measured against Ag/AgNO_3_. In the H-cell, the oxidation and reduction peaks of guaiacol lie between 0.91 V and 0.92 V, as well as 0.25 V and 0.3 V respectively. In the ATR-cell, the oxidation and reduction peak of guaiacol were measured between 1.01 V and 1.06 V, and at ∼0.8 V, respectively. Both CVs show qualitatively the same profile. The differences in distance between oxidation and reduction peak can be attributed to the different ion migration times between working and counter electrodes due to the different cell geometries. Fluctuation in the reference potential due to refilling of the reference electrode can add to the difference in half way potential (H-cell: 0.60 V; ATR-cell: 0.9 V).

The electrochemical performance of the custom-made cell was compared to a standard H-cell with 20 ml cell volume for each half cell equipped with Pt mesh for working and counter electrodes (see “*Ex situ* analytical methods” for details on the H-cell configuration). The CVs of both setups are shown Fig. 2 and show qualitatively identical features: a clear oxidation peak only in the presence of guaiacol in the potential window between 0.85 V and 1 V, and a minor reduction peak in the potential window between 0 V and 1 V *vs.* Ag^+^/AgNO_3_. These results indicate that the reaction behaviour is not significantly affected by the modified cell or electrode geometry and highlights the suitability of the custom cell for *in situ* spectroelectrochemical studies. The slight differences observed in the peak separation between the oxidation and reduction process are likely due to variations in ion migration times between the working and counter electrodes, caused by the markedly different cell geometries.^20^

### 3.2 Monitoring the oxidation of guaiacol

#### 3.2.1 *In situ* spectro-electrochemistry

**Fig. 3:**
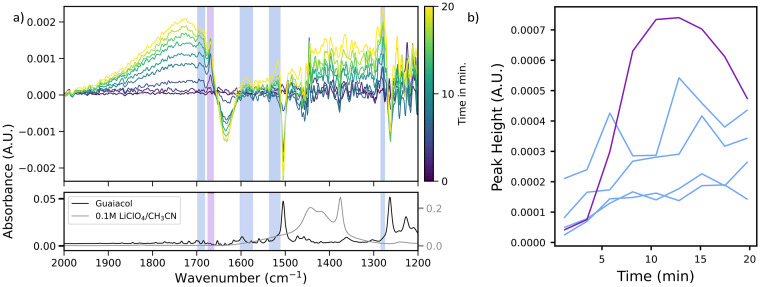
(a) Differential *in situ* attenuated total reflectance-infrared (ATR-IR) spectra of guaiacol electrooxidation over 20 min (top) and reference spectra of guaiacol and blank electrolyte (bottom), measured in the *in situ* cell with membrane. The first spectrum after applying the potential serves as the background. Negative bands indicate consumption, and positive bands formation of species. The negative bands at ∼1500 cm^−1^ and ∼1265 cm^−1^ can be clearly assigned to the *ν*(C=C)_aryl_, *ν*(Ar–O–C) vibrations of guaiacol. The decrease in absorbance of this band allows estimation of guaiacol’s conversion rate as 0.2 mM min^−1^. (b) Evolution of absorption band intensities over time for bands at ∼1668 cm^−1^ (violet) and at ∼1690, ∼1585, ∼1520, and ∼1280 cm^−1^ (blue).

Having established that the custom-built two-chamber electrochemical cell operates reliably and produces the same electrochemical events as a conventional H-cell, we applied it to study the electrochemical oxidation of guaiacol, a β-O-4 model compound. To account for potential shifts, such as those observed after refilling the reference electrode, the oxidation potential of guaiacol was determined prior to each experiment. This was achieved by performing cyclic voltammetry (CV) at low guaiacol concentrations, allowing the onset potential to be identified from the emergence of a positive peak in the recorded current. Subsequently, the electrolyte was replaced with a solution containing a higher guaiacol concentration, and chronoamperometry (CA) was conducted at a potential 0.05 V below the CV-determined onset to minimize the risk of overoxidation. Simultaneously, IR spectra were recorded at one-minute intervals. The data is displayed as differential absorbance spectra, using the first spectrum collected as right before the potential was turned on as background for all following spectra (Fig. 3a).

In interpreting the acquired spectra data, the background dynamics of the blank electrolyte without feedstock have to be taken into account. Control measurements recorded with blank electrolyte (*i.e.*, without guaiacol) showed minor but continuous spectral changes both under open-circuit conditions and during applied potential. These variations were observed particularly in the region ∼1620 cm^−1^ and between 1450 cm^−1^ and 1360 cm^−1^. We attribute these features to interactions between the LiClO_4_ salt, the acetonitrile solvent and trace amounts of water. If the reaction of interest shows features in these regions, they overlayed with the dynamics of the blank electrolyte. Therefore, they were treated with caution. Due to such regions of increased noise and since the changes observed happen in the order of mA.U., principal component analysis (PCA) was conducted for each measurement to ensure better differentiation between signal and noise (see ESI† for further details on the blank electrolyte measurements and the PCA analysis).

As Fig. 3a shows, during the first 20 min of electrochemical oxidation, two negative bands were observed at ∼1500 cm^−1^ and ∼1265 cm^−1^, respectively (see ESI† for the chronoamperometry measurements). The intensity of these bands decreased approximately linearly over time, suggesting continuous consumption of the starting material throughout the course of the reaction. A comparison with a reference spectrum of guaiacol allowed the assignment to the *ν*(C=C)_aryl_ vibration of the aromatic ring and *ν*(Ar–O–C) vibration of the ether linkage of guaiacol. Both vibrations are key spectral markers for guaiacol, and their gradual intensity decrease reflects the progressive breakdown of its aromatic and ether structures under oxidative conditions. By tracking the intensity change of these two bands, we were able to estimate a guaiacol consumption rate of ∼0.2 mM min^−1^ (see ESI† for further details on the quantification method).

Alongside the consumption of guaiacol, several new bands emerged over the course of the reaction, indicating the formation of electrochemical oxidation products. Notably, bands appeared at ∼1690 cm^−1^, ∼1585 cm^−1^, ∼1520 cm^−1^ and ∼1280 cm^−1^. Based on comparison with reference data and literature, the bands at ∼1690 cm^−1^, ∼1585 cm^−1^, and ∼1280 cm^−1^ fall within the characteristic regions of carbonyl-containing compounds, such as ketones. While the band at ∼1520 cm^−1^ is attributed to *ν*(C=C) stretching vibrations of multi-substituted aromatic systems.^21^ In addition, a sharp and well-defined band emerged at ∼1665 cm^−1^, which lies in the region typically associated with *ν*(C=O) vibrations of quinones as well as several ketone species, like diaryl ketones.^21^ This band exhibited a distinct temporal intensity profile compared to the others (Fig. 3b): the band at ∼1668 cm^−1^ rises sharply in intensity within the first 12 min of the reaction and then declines in intensity gradually thereafter.

Another noteworthy feature is the appearance of a broad IR band spanning from ∼1900 cm^−1^ and ∼1700 cm^−1^, with a maximum at ∼1725 cm^−1^. This band is exclusively observed during *in situ* measurements in the presence of guaiacol and is absent in both *ex situ* IR spectra of the extracted reaction products (see the “*Ex situ* product analysis” section) and in control experiments using blank electrolyte. This strongly suggests that the band arises from transient species or solvent–intermediate interactions that are only present under *in situ* conditions. A comparable feature was reported by Chen *et al.*,^22^ who also monitored the oxidation of simple β-O-4 model compounds in 0.1 M LiClO_4_/CH_3_CN using *in situ* IR spectroscopy. They observed similar broad absorption bands between 1850 cm^−1^ and 1660 cm^−1^ across their studied substrates. Specifically, these bands appeared in derivatives, such as 1-(4-ethoxyphenyl)ethanol, that contained either a phenolic group or an ethoxy substituent. In contrast, the structurally simpler 1-phenylethanol, which lacks both functional groups, did not exhibit this feature.

**Fig. 4:**
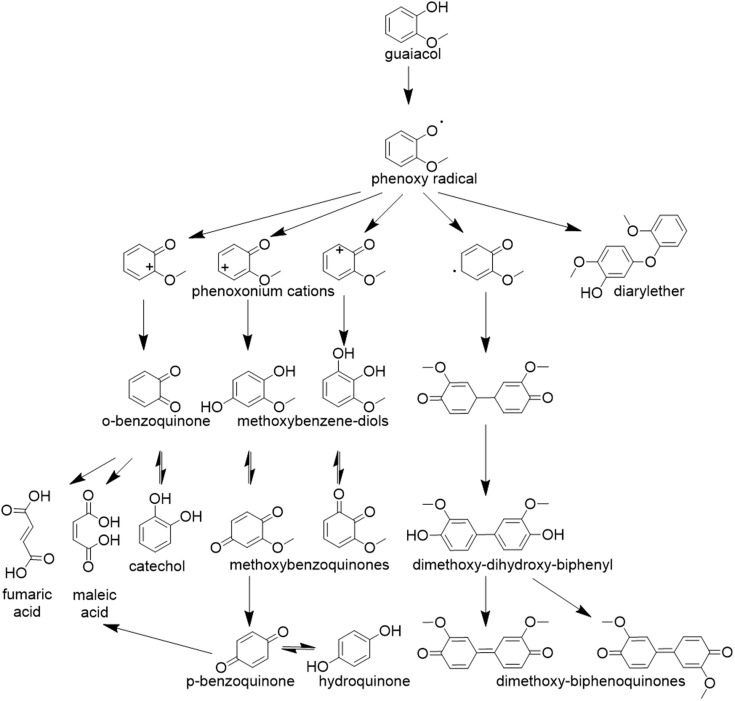
Guaiacol and its typical reaction pathway, which has been constructed from multiple literature sources.^13–17^

Chen *et al.* attributed the presence of these broad features to the formation of phenoxy radical intermediates and quinoid-type structures during the oxidation process. This assignment is in accordance with accepted reaction pathways for guaiacol oxidation to quinoids (see Fig. 4).^13,17^ The broad, unresolved nature of these features, potentially overlaid with sharper carbonyl-related bands, points toward dynamic solvation or conjugation effects involving reactive intermediates and the electrolyte environment.

Comparison with literature confirms that both quinones and substituted aromatics are well-established intermediates and products in the electrochemical and homogeneous catalytic oxidation of guaiacol.^13–17^ An overview of the most commonly suggested reaction pathways is provided in Fig. 4. Notably, the formation of a quinone *via* oxidation of the hydroxy group of guaiacol is consistently described as one of the first reaction steps in these pathways. This fits the temporal intensity behaviour of the band at ∼1668 cm^−1^ observed in our study, which we assigned to quinone-like species. Its sharp initial intensity rise followed by a gradual intensity decline likely reflects the transient accumulation and subsequent conversion of a carbonyl-bearing, quinone-like intermediate. Carboxylic acid formation, on the other hand, is generally associated with aromatic ring cleavage and further oxidation to maleic and fumaric acid. In contrast, if the ring and the methoxy group stay intact, methoxy benzoquinones or their corresponding diols are formed. Alternatively, cleavage of the methoxy group can lead to *p*-benzoquinone formation, which in turn may undergo further reaction to form catechol in the presence of residual water.

To further explore these possibilities and to confirm our band assignments, reference spectra of 0.1 M catechol and *p*-benzoquinone under the same conditions and electrolyte were recorded (see ESI† for further details). The catechol spectrum exhibits strong bands at ∼1585 cm^−1^, ∼1520 cm^−1^ and ∼1280 cm^−1^, which closely match features observed in the *in situ* ATR-IR spectroscopy measurements. Other catechol bands at ∼1469 cm^−1^, ∼1361 cm^−1^, and ∼1261 cm^−1^ are not observed in the *in situ* ATR-IR spectroscopy measurements. This can be rationalized, however, by the higher baseline noise between 1450 cm^−1^ and 1360 cm^−1^ due to electrolyte fluctuations, which may obscure weaker bands. Furthermore, the band at ∼1261 cm^−1^ is likely masked by the strong negative guaiacol ether band at the same position.

A reference spectrum of maleic acid was taken under the same conditions as those for catechol and *p*-benzoquinone, however the bands exhibited by maleic acid would all either overlap with the broad band between 1900 cm^−1^ and 1700 cm^−1^, or the bands inherent to the electrolyte at ∼1620 cm^−1^ and between 1450 cm^−1^ and 1360 cm^−1^. The reference spectrum of maleic acid was however compared to the *ex situ* IR spectrum of the extracted products (see the section “*Ex situ* product analysis”) where no match with maleic acid could be found.

The characteristic *ν*(C=O) stretching vibration of *p*-benzoquinone appears at ∼1660 cm^−1^, just below the observed quinone band at ∼1665 cm^−1^. The small shift could appear due to the overlay with the broad band between 1900 cm^−1^ and 1700 cm^−1^ and the negative band at ∼1636 cm^−1^, making *p*-benzoquinone another good potential fit for the observed band. However, *o*-benzoquinone would exhibit a band very close to that of *p*-benzoquinone. Theoretically, *ortho*- and *para*- benzoquinone could be told apart spectroscopically since the *para*-quinone has two bands in this region while the *ortho*-quinone only has one. However, the broad band from 1900 cm^−1^ to 1700 cm^−1^ prevents the observation of this tell tale band.^21^ In addition to these monomeric products, the formation of conjugated, dimeric or oligomeric species has been frequently reported in guaiacol oxidation studies. These species are typically associated with the development of a strong reddish-brown colour of the solution, which is a visual change that we also observed in our experiments, suggesting the likely presence of such coupling products.^14^

Taken together, these results support a mechanistic picture in which guaiacol undergoes initial oxidation *via* phenoxy radical formation, leading to quinoid intermediates. The transient broad band at ∼1725 cm^−1^ is likely a spectral fingerprint of this intermediate phase, while the appearance of stable bands associated with catechol and quinone derivatives further confirms their roles as key products in the reaction pathway. A summary of all assigned IR bands can be found in Table 1.

**Table 1:** Characteristic vibrational bands of guaiacol as well as possible products, corresponding to the experimentally observed absorption bands in the attenuated total reflectance-infrared (ATR-IR) spectroscopy data, obtained both *in situ* and *ex situ*

Molecule	Wavenumber (cm^−1^)	Vibration region
Guaiacol	1500	*ν*(C=C)_aryl_
	1265	*ν*(Ar–O–C)
Catechol	1585	*ν*(C=C)_aryl_
	1520	*ν*(C=C)_aryl_
	1280	*ν*(C–O)
	1200	*ν*(C–O)
Benzoquinone	1665	*ν*(C=O)_quin._
Methoxy-*p*-benzoquinone	1680	*ν*(C=O)
	1665	*ν*(CO)_quin._
	1592	*ν*(C=C)_aryl_
	1240	*ν*(Ar–O–C)
Ketone species	1690	*ν*(C=O)

#### 3.2.2 *Ex situ* product analysis

To complement our *in situ* ATR-IR spectroscopy observations, guaiacol was electrochemically oxidized in a conventional H-cell and the resulting products were extracted and analysed as outlined in the Experimental section. Each *ex situ* measurement was compared to the results of an extraction of the unreacted feedstock mixture, 0.1 M guaiacol in 0.1 M LiClO_4_ in acetonitrile. The spectrum of the extracted reaction mixture is dominated by the unreacted guaiacol bands, indicating limited conversion (see ESI† for further details). To isolate signals originating from the reaction products, the spectrum of the unreacted feedstock was subtracted. A multitude of bands emerge, consistent with the presence of multiple compounds, as would be expected from guaiacol oxidation (see ESI† for further details).^3^

**Fig. 5:**
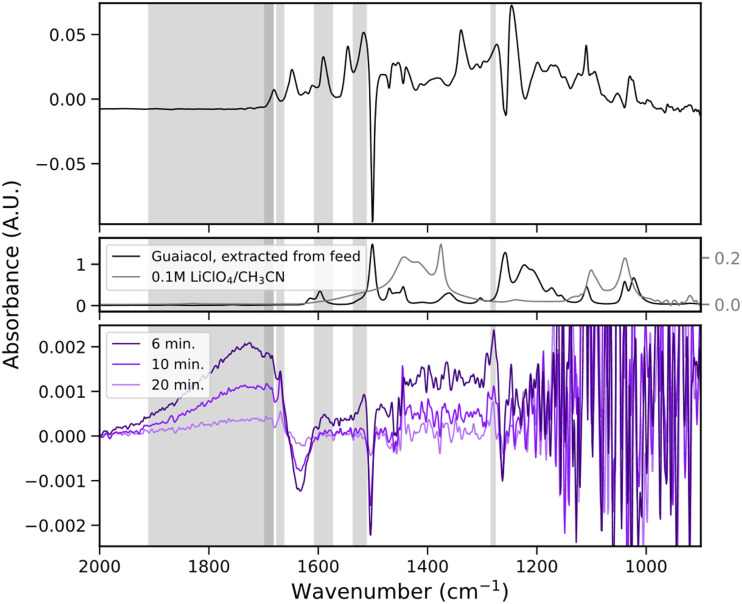
Attenuated total reflectance-infrared (ATR-IR) spectra showing (top) the difference between the extracted product and feedstock spectra, with reference spectra of guaiacol and the electrolyte below. (Bottom) Differential *in situ* ATR-IR spectra of guaiacol oxidation at three time points over 20 min. Significant bands from the *in situ* ATR-IR data are shaded in grey across all plots for comparison.

When compared to the *in situ* ATR-IR spectra, several familiar bands reappear in the *ex situ* IR spectra, reinforcing the presence of consistent molecular features across both measurement techniques (Fig. 5). Notably absent, however, is the broad absorption band spanning the 1900–1700 cm^−1^ region. This supports the earlier hypothesis that the broad band observed in *in situ* ATR-IR spectroscopy measurements is associated with transient intermediates, such as the phenoxy radical, or solvation effects involving the electrolyte, that are lost upon workup and extraction.

To investigate potential carboxylic acid products, the *ex situ* spectrum was overlaid with a reference spectrum of 0.1 M maleic acid recorded in the ATR-IR *in situ* cell. The characteristic maleic acid band at ∼1739 cm^−1^ is absent in the product extract, suggesting that maleic acid is not present in significant quantities. On the other hand, there is a strong spectral match with both benzoquinone and catechol, whose reference spectra align well with the observed features (see ESI† for further details). The references of *p*-benzoquinone and catechol were also recorded at 0.1 M in the electrolyte filled *in situ* cell. Minor discrepancies in band positions can be attributed to artifacts from the subtraction of the guaiacol bands, which are especially pronounced at guaiacol’s strongest bands at ∼1500 cm^−1^ and ∼1265 cm^−1^ and overlapping signals from other components in the product mixture, which can be expected for the band at ∼1648 cm^−1^.

To extend the analysis beyond easily accessible reference molecules, a digitalized reference spectrum of methoxy-*p*-benzoquinone, sourced from literature^23^ and processed using WebPlotDigitizer (version 4, automeris.io), reveals another convincing match, particularly for not accounted for by catechol or benzoquinone. It should be noted, however, that the band at ∼991 cm^−1^ is absent in our spectrum as this is beyond the optical transparency of the used Si ATR crystal. A similar increase in noise is observed in this region for spectra recorded of *p*-benzoquinone and catechol (see ESI† for further details). The partial overlap of methoxy-*p*-benzoquinone bands with those of *p*-benzoquinone and catechol at 1647 cm^−1^ and 1590 cm^−1^ can also explain the differences between the observed bands and the references in the *in situ* spectra. The band assignments are summarized in Table 1. Corresponding NMR and TLC measurements also indicated limited conversion. In the NMR spectra, the guaiacol signal was so predominant that few other signals could not be distinguished, although the presence of oligomers was confirmed. Further NMR analysis would require separation of the products from unreacted guaiacol. However, TLC results show that separation is challenging; even with the most effective eluent mixture tested (hexane/benzene/acetone, 40 : 30 : 30), no clear separation was achieved.

### 3.3 Influence of cell design: membrane *vs.* no membrane

**Fig. 6:**
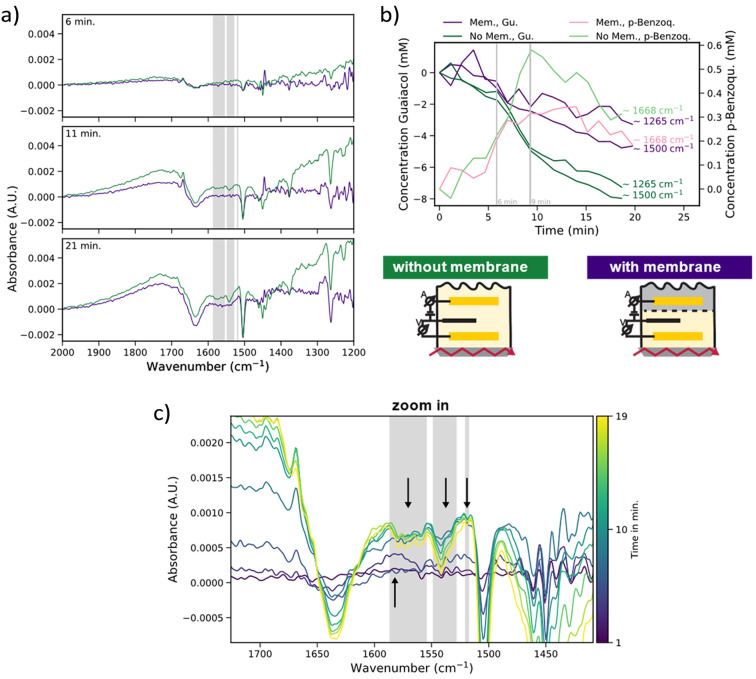
(a) Differential *in situ* ATR-IR spectra measured with (violet) and without (green) a membrane at three time points. Grey shading highlights three decreasing bands at ∼1570 cm^−1^, ∼1542 cm^−1^, and ∼1519 cm^−1^, which appear only without the membrane. (b) Conversion of guaiacol and the quinone species observed in its absorption band at ∼1668 cm^−1^. The quinone species concentration was estimated with Lambert–Beer’s law from the absorption measured in the *in situ* measurements at ∼1668 cm^−1^ and the absorption at ∼1660 cm^−1^ measured for 0.1 M of *p*-benzoquinone. (c) Zoom into the 1750–1350 cm^−1^ region of the attenuated total reflectance-infrared (ATR-IR) spectra without membrane, showing the same decreasing bands (grey) and an increasing band at ∼1580 cm^−1^ that becomes overlapped by the ∼1570 cm^−1^ band as the reaction progresses.

To assess the impact of the half-cell separation on the spectroscopic results, additional *in situ* IR spectroscopy experiments were performed using a single-chamber configuration, *i.e.*, without membrane between the working and counter electrode, as typically performed in literature. The resulting IR spectra at different reaction times are compared to those obtained with the two-chamber, membrane-separated cell, as shown in Fig. 6a.

The features shared between the ‘with’ and ‘without membrane’ spectra are readily evident. Most notably, the electrochemical conversion of guaiacol is evident in each setup, as indicated by the progressive intensity decrease of the characteristic guaiacol bands at ∼1500 cm^−1^ and ∼1265 cm^−1^. Simultaneously, both setups exhibit increasing absorption band intensities at ∼1690 cm^−1^, ∼1585 cm^−1^, ∼1520 cm^−1^, and ∼1280 cm^−1^, features that mirror those assigned earlier to aromatic, and ketonic products and intermediates, such as catechol and quinones, which all display a similar reaction rate as in the membrane-separated case. Additionally, the distinct band at ∼1668 cm^−1^ appears in both cell design configurations, showing the same dynamic behaviour: a rapid rise during the initial 10 min followed by a gradual decline. Finally, both cell design configurations also show the broad band between 1900 cm^−1^ and 1700 cm^−1^. These overlapping features indicate that, regardless of whether a membrane is used, guaiacol oxidation proceeds through a broadly similar reaction pathway. The consistent presence of quinone related bands in both experiments suggests that diffusion between the electrode compartments does not fundamentally alter the core chemistry.

However, several decreasing bands appear uniquely in the IR spectra recorded without a membrane, pointing to side reactions or additional intermediates enabled by the absence of a diffusion barrier. A broad band centred at ∼1570 cm^−1^, a sharper band with a shoulder towards lower wavelength at ∼1542 cm^−1^ and another weak, but sharp band at ∼1519 cm^−1^ (shaded grey in Fig. 6a and c). These bands are not present in the membrane-separated system, indicating that their formation may be linked to secondary reactions or re-reduction involving species that diffuse freely between the electrodes.

These differences in cell configuration are further reflected in the reaction kinetics (Fig. 6b). Using Lambert–Beer’s law, quinone concentration was estimated from its absorbance at ∼1668 cm^−1^ and the absorbance measured for the 0.1 M *p*-benzoquinone reference at ∼1660 cm^−1^. In both cells, the conversion of guaiacol proceeds approximately linearly. However, while the initial reaction rates are comparable for the first 6 min, the cell without membrane displays a pronounced rate increase between 6 and 9 min, followed by a decline. This acceleration coincides with the maximum observed quinone concentration, suggesting transient accumulation and subsequent depletion or transformation of intermediates. In contrast, the cell equipped with a membrane shows a subtle change in conversion rate around minute 7, which also roughly corresponds to the peak quinone concentration in this configuration. However, the maximum conversion rate achieved in this setup is lower than that of the cell without membrane. When interpreting these kinetic differences, the instability of the oxidation current in the membrane-separated cell must be considered. Nevertheless, the influence of differing cell configurations due to the possibility of additional reactions occurring at the counter electrode in the membrane-less system cannot be excluded.

A closer inspection of the region between ∼1606 cm^−1^ and ∼1559 cm^−1^ revealed an increasing band intensity at ∼1580 cm^−1^, which appears to get overlayed by the decreasing band at ∼1570 cm^−1^ after ∼10 min (Fig. 6c). The bands all lie in the region of *ν*(C=C), *ν*(C–C) and *ν*(C=O) vibrations. Taken together, the spectral evolution suggests the formation of differently substituted aromatic systems. These likely result from the back-reduction of quinone intermediates under conditions of unimpeded species crossover or different reaction selectivity in the absence of a membrane.

Together, these findings underscore the importance of proper cell design in *in situ* electrochemical studies. While both configurations support core mechanistic observations, the presence or absence of a membrane can significantly influence reaction pathways and intermediate lifetimes.

## 4. Conclusion

A novel cell design for an electrochemical cell enabling *in situ* attenuated total reflectance-infrared (ATR-IR) spectroscopy was developed and applied to investigate the electrochemical oxidation of the lignin monomer guaiacol. This ATR-IR cell design facilitates spectro-electrochemical measurements compatible with corresponding H-cell measurements alongside IR spectroscopy. The results obtained from guaiacol oxidation align well with findings reported in the literature, as indicated by the appearance of characteristic IR fingerprint bands corresponding to expected species and reaction intermediates, such as *p*-benzoquinone and catechol. These findings were further supported by *ex situ* IR spectroscopy analysis of the reaction products extracted from parallel experiments performed in an H-cell under identical conditions, apart from differences in the cell and electrode geometry. The *ex situ* IR spectroscopy experiments also give a good indication of the presence of methoxy-*p*-benzoquinone, another of the expected guaiacol oxidation products, which could also be weakly present in the *in situ* recorded ATR-IR spectra.

Furthermore, we could show that the absence of a diffusion barrier between the working and counter electrodes leads to decreasing intensities of the vibrational bands in the spectral regions associated with *ν*(C=C), *ν*(C–C) and *ν*(C=O) vibrations between 1600 cm^−1^ and 1520 cm^−1^, which are not present if a membrane is installed. This observation underscores the potential for misleading results when using simpler cell configurations, particularly in complex reactions. Therefore, employing a more refined cell design that minimizes unintended reaction pathways is crucial for accurate spectroscopic data interpretation.

The promising outcomes of this study highlight the need for further refinement of the developed experimental setup, especially in relation to maintaining stable potentials and currents during reactions known to passivate the electrocatalyst material, an issue that becomes particularly pronounced in the small volumes typical of *in situ* cells. Additionally, supplementary experiments not detailed here pointed to the importance of optimizing the counter reaction to enable extended operation times for the *in situ* cell constructed.

## Author contributions

The manuscript was written through contributions of all authors. All authors have given approval to the final version of the manuscript. Conceptualization: BMW, SMKS and BB; methodology: SMKS, SD and BB; data curation: SMKS; formal analysis, investigation: SMKS (lead) and BB (contributed); writing – original draft: SMKS; writing – review & editing: BB and BMW; supervision: BB and BMW; funding acquisition: BMW; discussion support: MB and SD.

## Conflicts of interest

The authors declare that they have no known competing financial interests or personal relationships that could have appeared to influence the work reported in this paper.

## Supplementary Material

FD-263-D5FD00069F-s001

## Data Availability

All data and python scripts utilized in the manuscript have been uploaded to the YODA repository. The DOI to access the data is https://doi.org/10.24416/UU01-3U20Y2.
