# Supplementary material for: Spectro-electrochemistry of guaiacol oxidation: tracking intermediates in a membrane-separated cell with in situ attenuated total reflectance-infrared spectroscopy
Source: Faraday Discuss. 2025 Oct 27;263:164–81. doi: 10.1039/d5fd00069f (PMC12557300; doi:10.1039/d5fd00069f)
Supplement: FD-263-D5FD00069F-s001 [file FD-263-D5FD00069F-s001.pdf]

Supplementary Information (SI) for Faraday Discussions.
This journal is © The Royal Society of Chemistry 2025

Spectro-Electrochemistry of Guaiacol Oxidation: Tracking Intermediates in a Membrane-Separated Cell with In Situ Attenuated Total Reflectance-Infrared Spectroscopy

Sibylle M. K. Schwartmann,[a] Mariangela Biggiero,[a] Bettina Baumgartner,[a,b,*] Bert M. Weckhuysen[a,*] and Ing. Sander Deelen [a]

[a]Inorganic Chemistry and Catalysis group, Institute for Sustainable and Circular Chemistry, Utrecht University, Universiteitsweg 99, 3584 CG Utrecht, Netherlands

[b]Van 't Hoff Institute for Molecular Sciences, University of Amsterdam, Science Park 904, 1098 XH Amsterdam, Netherlands

Email: *b.m.weckhuysen@uu.nl, *b.baumgartner@uva.nl

Table of Contents

Table of Contents 1

1. Permeability Test Protocol for Membranes 2
2. Analysis of In Situ Spectra 2
   - 2.1. Time Dependent Spectral Features of the Blank Electrolyte 2
   - 2.2. Principle Component Analysis (PCA) of Differential In situ Spectra Recording Guaiacol Electrooxidation 3
3. Chronoamperometry Data 4
4. Assessment of Absorbance over Time 6
   - 4.1. Estimation of Guaiacol Consumption Rate 6
5. Ex Situ Infrared Analysis of the Extracted Products 7

1. Permeability Test Protocol for Membranes

The attenuated total reflectance (ATR) cell was assembled by filling both the top and bottom chambers with blank electrolyte. Following equilibration, a background spectrum was recorded, after which guaiacol was introduced into the top chamber. Membrane performance was evaluated by monitoring analyte diffusion across the separator using the technique of ATR-infrared (IR) (further denoted as ATR-IR) spectroscopy. Permeable membranes, such as Fumasep FAA-3-PK-130, allowed guaiacol to migrate into the bottom chamber, as evidenced by the appearance of characteristic vibrational bands. In contrast, Nafion 117, a non-permeable membrane under these reaction conditions, exhibited no detectable guaiacol-associated signals over a 24 h diffusion period. To ensure sufficient ionic conductivity, the Nafion membrane was lithiated and re-evaluated. While enabling Li^+ transport, the lithiated Nafion remained impermeable to guaiacol.

2. Analysis of In Situ Spectra

2.1. Time Dependent Spectral Features of the Blank Electrolyte

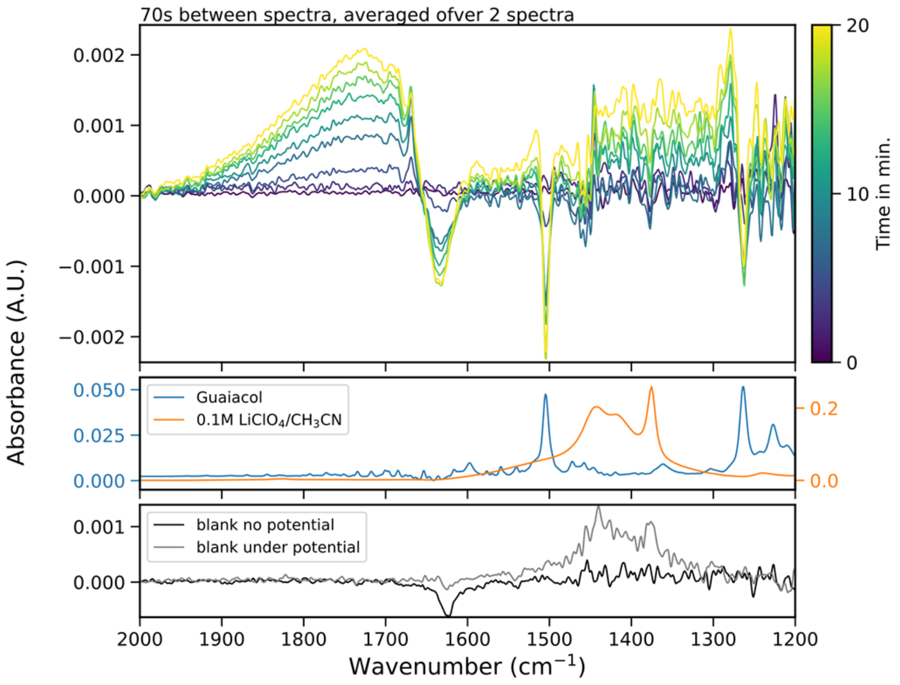

Figure 1 (Top) Differential in situ attenuated total reflectance-infrared (ATR-IR) spectra of the electro-oxidation of guaiacol during 20 min of reaction. (Middle) Reference spectra of 0.1 M guaiacol and blank electrolyte. (Bottom) final spectra after 20 min of chronoamperometry at 0.9 V and without a potential applied. All measurements were conducted in the in situ ATR-IR spectro-electrochemical cell, while the Li^+-Nafion 117 membrane was installed. The blank electrolyte showed emerging spectral features in the regions at ~ 1620 cm^{-1} and between 1450 cm^{-1} and 1360 cm^{-1}. The band decreasing at ~ 1620 cm^{-1} lies in the region of $\delta(OH)$ bending vibrations, propably due to changes of the water impurites in the acetonitrile. The band between 1450 cm^{-1} and 1360 cm^{-1} is at the same position and mirrors the shape of the broad band in aceotnitrile.

2.2. Principle Component Analysis (PCA) of Differential In situ Spectra Recording Guaiacol Electrooxidation

The principal components (PCAs) were calculated with the sklearn python module.

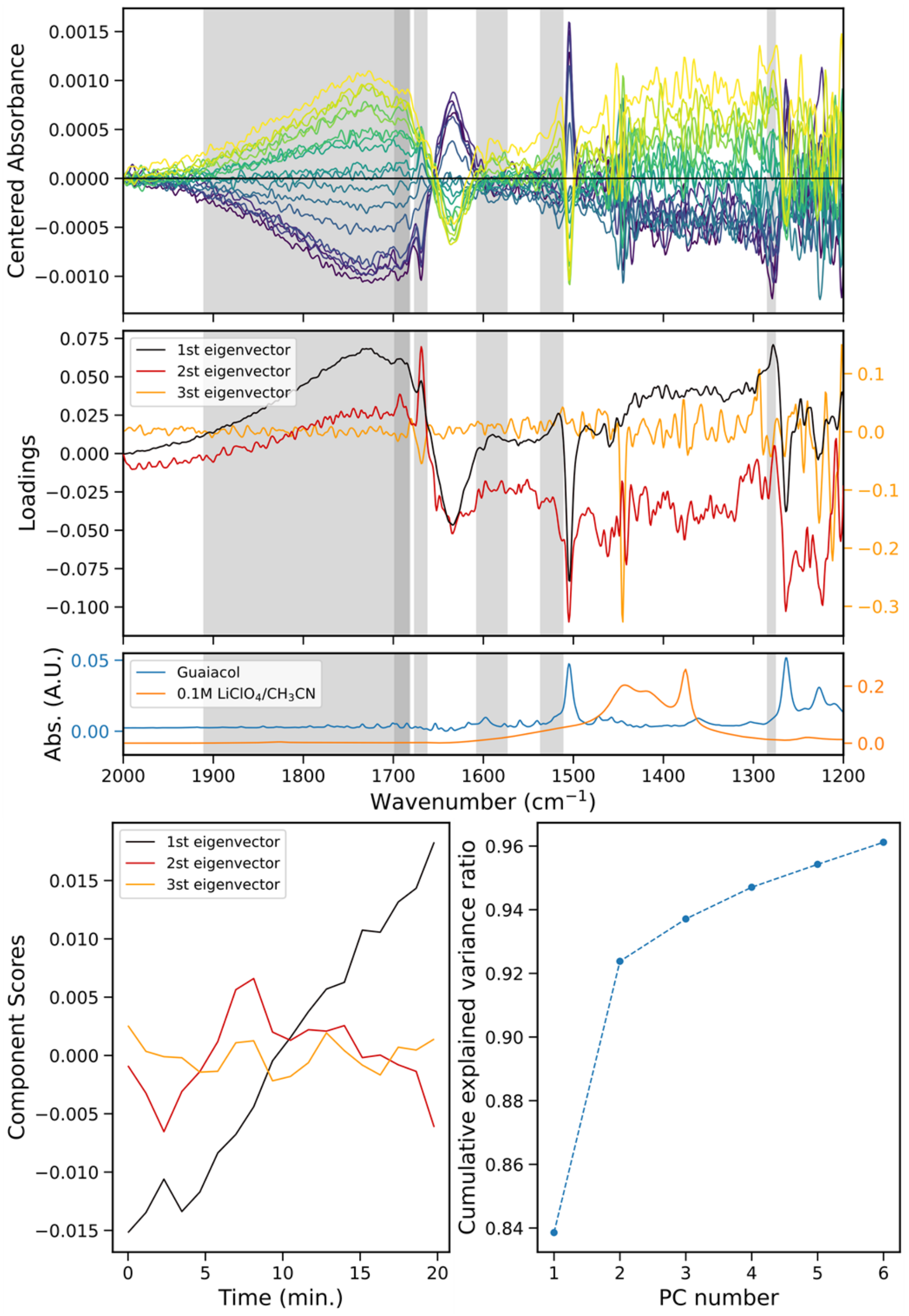

Figure 2 Principal Component Analysis (PCA) of differential in situ attenuated total reflectance-infrared (ATR-IR) spectra recorded during the electro-oxidation of guaiacol. (Top to bottom of top figure) Centered Absorbance as it is processed by the PCA; the first 3 components of the PC; reference spectra of guaiacol (blue) and electrolyte (orange). (Bottom figure, left) Scores of the first three components vs. oxidation time. (Bottom, right) Cumulative explained variance for the first 6 components.

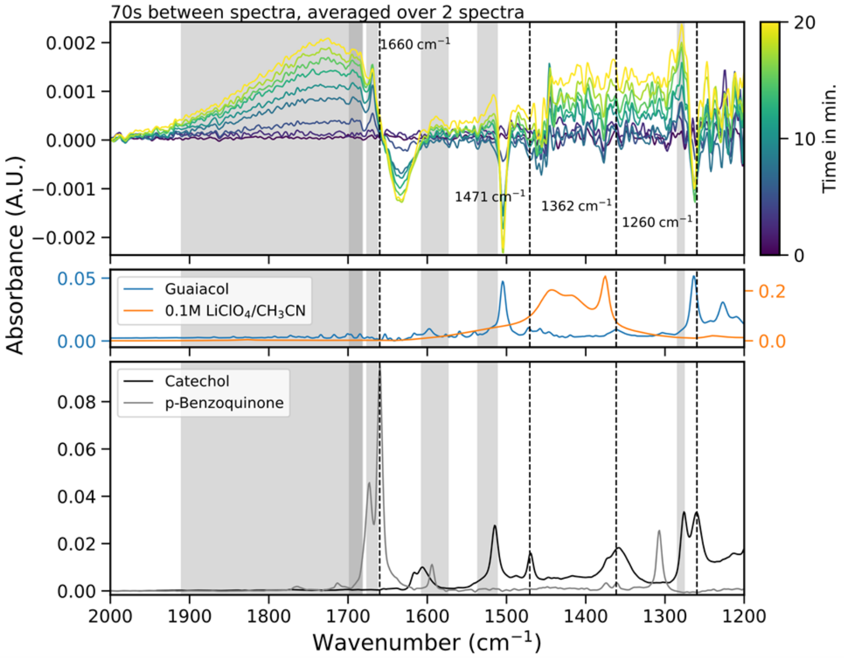

Figure 3 From top to bottom: In situ IR spectra obtained during 20 min of guaiacol electrooxidation, References of guaiacol (blue) and electrolyte (orange) taken in the cell, reference measurement of 0.1 M catechol taken in the electrolyte (black) and reference measurement of 0.1 M p-benzoquinone in electrolyte (grey). The strong catechol band shows a good match with the bands observed in situ at 1585 cm^{-1}, 1520 cm^{-1} and 1280 cm^{-1}. The catechol bands at 1469 cm^{-1}, 1361 cm^{-1}, and 1261 cm^{-1} are not observed but they might not be discernable from the heavier noise in the region from 1469 cm^{-1} to 1361 cm^{-1}. The band at 1668 cm^{-1} is a good fit to the $\nu(C=O)_{quin}$ vibration of p-benzoquinone or o-benzoquinone if one accounts for the shift to higher wavenumbers the overlay with the broad band between 1900 cm^{-1} and 1700 cm^{-1} and the negative band at 1636 cm^{-1} due to the electrolyte fluctuations would cause but a fit.

3. Chronoamperometry Data

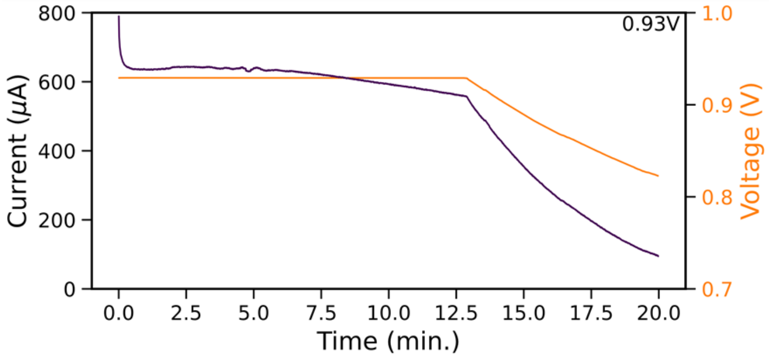

Figure 4 Chronoamperometry obtained during the oxidation of guaiacol over 20 min, as recorded in the in situ attenuated total reflectance-infrared (ATR-IR) cell with a membrane installed. The potentiostat was set to continuously apply 0.93 V. However, the record shows that the potential starts a sudden continuous decrease after 13 min of reaction, which is accompanied by a sudden start in current decline at the same time.

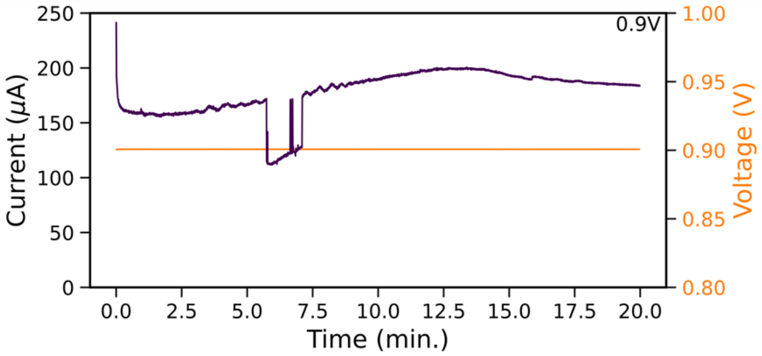

Figure 5 Chronoamperometry to oxidize guaiacol over 20 min, as recorded in the in situ attenuated total reflectance-infrared (ATR-IR) cell without a membrane installed. The potentiostat was set to continuously apply 0.9 V, and this potential was kept uninterrupted throughout the whole measurement and the current does not show a sudden decline as well.

We attribute the unstable electrode potentials to the formation and deposition of oligomers or dimers, generated through condensation of electrochemical intermediates, on critical conductive components, such as the working electrode. A reddish-brown residue was consistently observed on both the working electrode and the membrane following each experiment. Notably, this potential instability was exclusive to the measurements conducted in the membrane-separated ATR cell. In contrast, the H-cell experiments and ATR-IR spectroscopy configurations without a membrane exhibited a stable potential throughout the whole measurement duration. These observations suggest that electrode passivation occurs more rapidly in the compartmentalized ATR-IR setup, likely due to the reduced cell volume. The confined geometry of the membrane-separated cell, in comparison to the larger volume of the H-cell or the expanded single-chamber configuration without a membrane, may accelerate local concentration build-up and deposition processes, thereby promoting faster passivation of the working electrode or membrane interface.

4. Assessment of Absorbance over Time

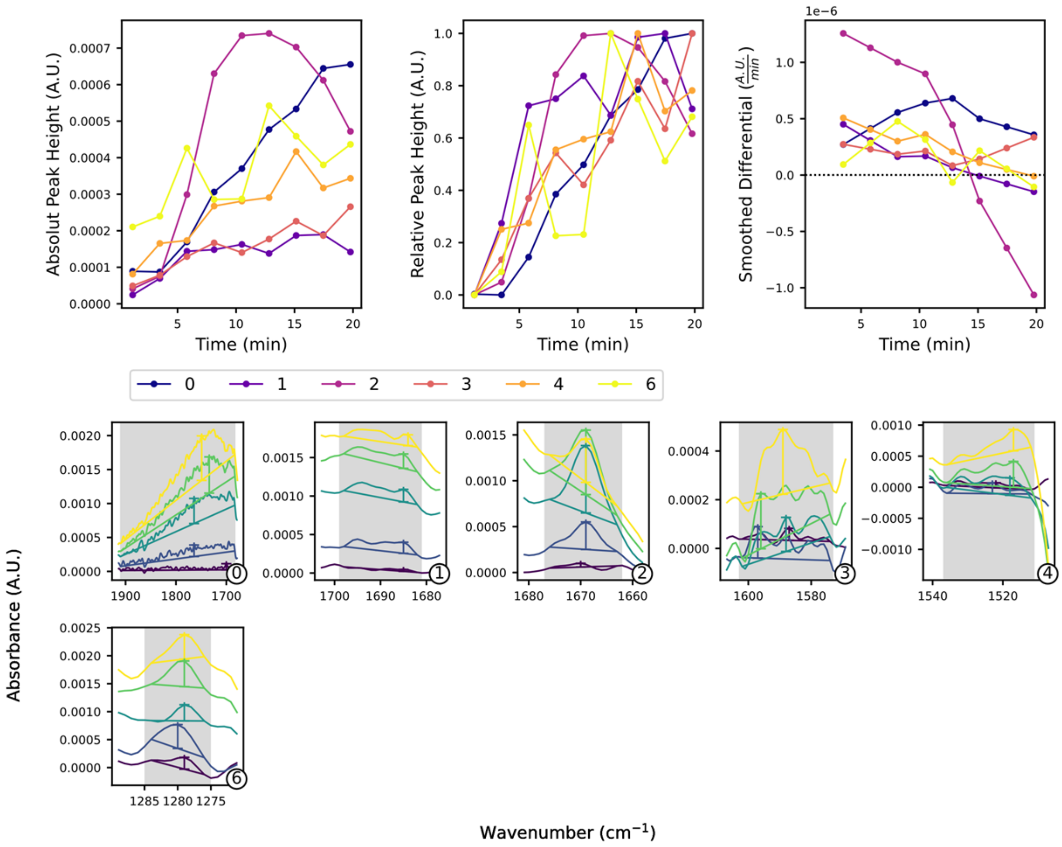

Figure 6 (Top) Temporal evolution of peak intensities for all observed increasing bands during electrochemical oxidation as measured with in situ attenuated total reflectance-infrared (ATR-IR) spectroscopy. (Bottom) Methodology for absorption band height determination. Grey-shaded regions indicate the wavenumber ranges in which each absorption band appears. For each spectrum, a linear baseline was estimated by connecting absorbance values at the flanking high- and low-wavenumber edges of the band. Peak height was then quantified as the vertical distance from this baseline to the band maximum at each time point.

4.1. Estimation of Guaiacol Consumption Rate

The intensities of the absorption bands of the $\nu(C=C)_{aryl}$ and $\nu(Ar-O-C)$ vibration (located at ~ 1500 cm^{-1} and 1265 cm^{-1}, respectively) is measured in the same manner as outlined above for the time dependent measurements and as shown in Figure 7 for the reference spectrum of guaiacol. To calculate an estimation of the guaiacol concentration at each timestep, Beer-Lambert's law has been used: $A = \epsilon d_e C$. A is the absorbance, ϵ is the absorptivity of guaiacol, and d_e is the optical path length. All values are wavenumber dependent. Using the absorbance measured of the guaiacol reference at 0.1 M in the electrolyte we get an estimate for the guaiacol conversion rate over time, as shown in Figure 9 for the configuration with chamber separation (left) and without (right).

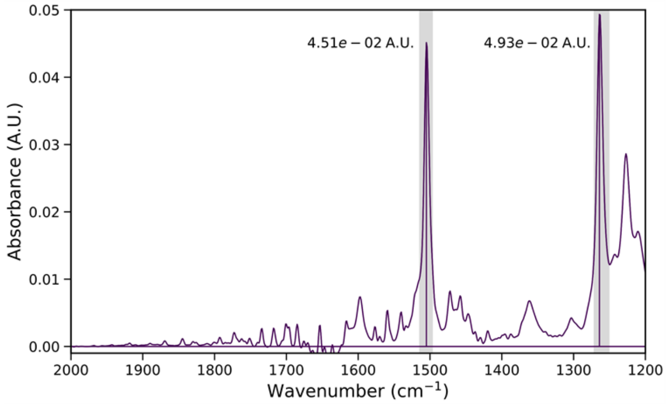

Figure 7 Attenuated total reflectance-infrared (ATR-IR) spectrum of 0.1 M guaiacol in 0.1 M $LiClO_4$ in acetonitrile recorded in the in situ ATR-IR cell with electrolyte as background. The residual noise is due to water vapor present in the sample compartment of the spectrometer. The absorbance of the band at ~ 1500 cm^{-1} and ~ 1265 cm^{-1} were measured from zero and determined to be 4.51 · 10^{-2} A.U. and 4.93 · 10^{-2} A.U., respectively.

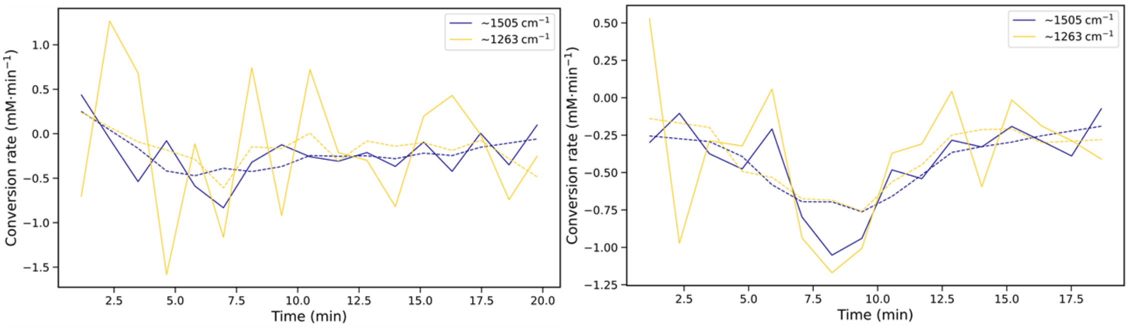

Figure 8 Conversion rate of guaiacol over time as measured in the attenuated total reflectance-IR (ATR-IR) cell with (left) and without (right) membrane separation. Dotted lines correspond to the data smoothed using the Savitzky-Golay filter (scipy savgol_filter, window length: 5, poly. order: 1), included to guide the eye.

5. Ex Situ Infrared Analysis of the Extracted Products

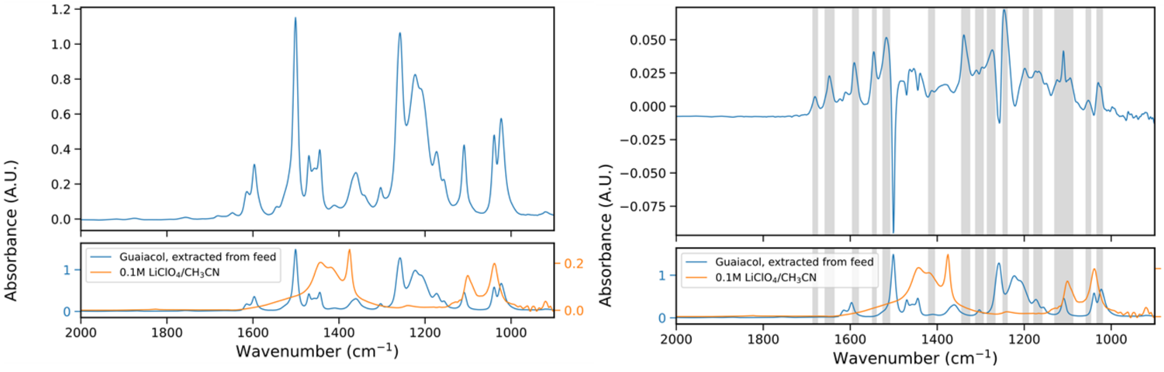

Figure 9 (Left) Attenuated total reflectance-infrared (ATR-IR) spectrum of the reaction products extracted from the oxidation of guaiacol in an H-cell is shown in the top plots, below are the ATR-IR spectra of the extracted unreacted feedstock (i.e., guaiacol, extracted from feed) and the electrolyte as reference (0.1 M/$LiClO_4$/CH_3CN). (Right) IR spectrum of the extracted products after subtracting the spectrum of extracted unreacted feedstock multiplied by an adjusting factor. Pre-subtraction the spectrum is dominated by the guaiacol signal, showing that the oxidation only yielded limited conversion. After subtraction a multitude of bands, marked in grey, become visible as would be fitting for a mixture of compounds expected from the oxidation of lignin model compounds.

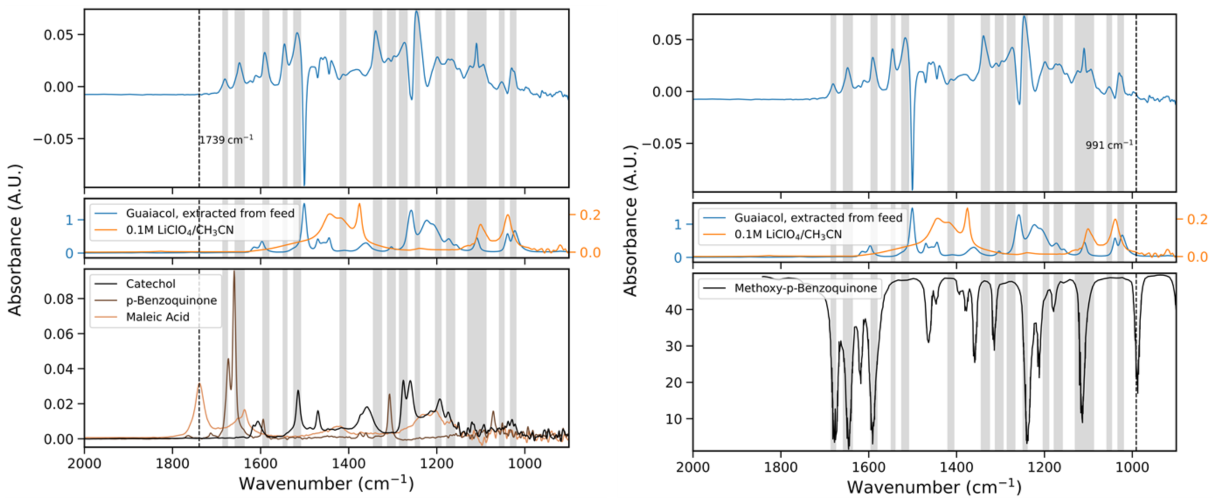

Figure 10 Attenuated total reflectance-infrared (ATR-IR) spectroscopy data resulting from the difference between the IR spectrum of the extracted products and the extracted feedstock in the top plot, with reference spectra of the feedstock guaiacol and the electrolyte underneath. Both plots have the bands from the product mixture shaded in grey. (Left) Spectrum of the final product mixture compared to reference spectra below of catechol, p-benzoquinone and maleic acid, each taken at 0.1 M electrolyte. (Right) Spectrum of the final product mixture compared to a transmission spectrum of methoxy-p-benzoquinone taken from [23].
